# A Narrative Review of Men’s Mental Health: The Role of Stigma and Gender-Differentiated Socialization

**DOI:** 10.3390/bs16020262

**Published:** 2026-02-11

**Authors:** Julio A. Camacho-Ruiz, Carmen M. Galvez-Sánchez, Rosa M. Limiñana-Gras

**Affiliations:** 1 Department of Personality, Evaluation and Psychological Treatment, Faculty of Psychology and Speech Therapy, University of Murcia, Building 31, 30100 Murcia, Spain; julioangel.camachor@um.es (J.A.C.-R.); liminana@um.es (R.M.L.-G.); 2 Foundation Project Man Jaén, 23002 Jaén, Spain; 3 Regional International Campus of Excellence (CEIR) Mare Nostrum Campus (CMN), 30100 Murcia, Spain; 4 Assisted Reproduction Unit, QuironSalud Murcia Medical Center, 30008 Murcia, Spain

**Keywords:** men, mental health, stigmas, gender, gender-differentiated socialization

## Abstract

Research on men’s mental health points out gender differences in help-seeking and access to care. Traditional masculine norms (i.e., emotional repression, self-reliance, “*being strong*”) and gender bias might conceal distress, delay treatment, and help to explain higher burdens of addiction, violence, and suicide alongside lower recorded affective/anxiety diagnoses. An exploratory narrative review was conducted. PubMed, Scopus, and Web of Science were searched for 2015–2025 studies using MeSH and terms on men’s mental health, masculinities, and stigma. Eleven studies identified attitudinal barriers (i.e., self-stigma, shame, symptom minimization, mistrust, etc.) and structural barriers (i.e., limited tailored services, navigation difficulties, costs, bureaucracy, etc.) that delay identification of psychological distress symptoms, weaken therapeutic alliance, and increase dropout, especially when therapy is perceived as impersonal or ineffective. Intersectional factors (i.e., class, age, ethnicity) further contribute to access and they need to be included in the field of men’s mental health. Gender-sensitive approaches and alternative masculinity role models have the potential to enhance engagement and legitimize emotional experience. To sum up, hegemonic masculinity-related gender norms, acquired through gender-differentiated socialization, are associated with adverse mental health outcomes among men. A lack of gender-sensitive awareness campaigns to reduce stigma around men’s mental health may hinder prevention, delaying early identification and timely intervention. Therefore, men’s mental health care should integrate gender and intersectionality transversally to improve prevention, access, diagnosis, treatment, adherence, and outcomes, supported by professional training and tailored therapeutic tools in clinical routine practice. These findings underscore the need to promote healthier, more egalitarian masculinities and to deconstruct stigmas associated with help-seeking and mental health service.

## 1. Introduction

### 1.1. Men’s Mental Health in Context: Masculinity Norms and Access to Care

At present, the available evidence indicates a growing body of research on men’s mental health, alongside studies that aim to identify and characterize gender-related differences in help-seeking behaviors and access to mental health services and treatment (Addis, 2008; Oliffe & Han, 2014; Seidler et al., 2022). These findings are, in a large number of cases, directly related to differences in the expression of emotional distress among men. They are also linked to access to the healthcare system when data are compared with women’s access to mental health treatment resources. Therefore, emphasis is placed on the relevance of making men’s emotional distress visible and on creating intervention perspectives that also focus on psychosocial issues such as gender, which may influence men’s access to treatment (Addis, 2008; Oliffe & Han, 2014; Seidler et al., 2022). Accordingly, the objective of this narrative review is to examine how hegemonic masculinity norms and gendered socialization processes shape attitudinal and structural barriers to men’s access to mental health resources and engagement with care, as well as to outline gender- and intersectionality-informed therapeutic strategies to enhance treatment adherence and outcomes.

The gender perspective in the treatment of men with mental health-related problems is positioned as a highly relevant methodological approach. This treatment perspective might help to address and understand the existing differences in the ways in which problems derived from emotional and psychological suffering are approached in men, as well as in the field of health more broadly. The aim is to incorporate gender transversally as a key indicator when intervening with men, understanding that it directly influences the subjective construction of men’s identities (Connell & Messerschmidt, 2005; Courtenay, 2000; Kimmel, 2019).

A gender perspective in mental health allows for the examination of how gender norms and mandates shape the experience of psychological distress and individuals’ relationships with mental health services. In the case of men, traditional masculinity norms, such as self-reliance, emotional control, and the avoidance of vulnerability, have a significant influence on symptom identification, help-seeking behaviors, and the therapeutic relationship (Addis & Mahalik, 2003). Incorporating this perspective into research and treatment involves critically examining these mandates and adapting interventions to reduce access barriers, promote emotional expression, and strengthen the therapeutic alliance.

A significant portion of the existing research related to men’s mental health makes it necessary to reflect on a highly contradictory issue: on the one hand, data show that, compared with women, men are predominant in treatments for addictive disorders, in completed suicides, and in the more frequent perpetration of violence. On the other hand, data indicate that diagnoses of affective or anxiety disorders are considerably lower among men than among women (Scourfield et al., 2012; WHO, 2018). This contradiction could potentially be explained by the fact that more instruments are available to diagnose affective or anxiety disorders, which are more common among women, than instruments designed to identify aggressive behaviors, stress, or introverted behaviors, which are frequent among men (Brownhill et al., 2005; Rice et al., 2018a).

Within this framework, the work of Stephen Frosh offers a key contribution by articulating the social, narrative, and clinical levels of masculinity. From a psychosocial and psychoanalytic perspective, Frosh conceptualizes masculinity as a fragile achievement—shaped by defenses and contradictions—rather than as a coherent and stable identity (Frosh, 1994, 1995). His analyses show how mechanisms such as homophobia, the rigidification of rationality, or subjective fragmentation operate as defensive strategies in the face of the threat of vulnerability (Frosh, 1992; Frosh et al., 2005). These dynamics are particularly relevant in the field of mental health, where they may translate into resistance to the therapeutic relationship, difficulties in mentalizing distress, and ambivalence towards dependence on professional care. Consequently, integrating a perspective on gender and subjectivity into mental health interventions is essential for understanding the specific obstacles many men face and for promoting clinical practices that are more attuned to the relational, emotional, and symbolic dimensions of masculinity (Frosh et al., 2017).

Moreover, this contradiction may also be attributable to gender-related biases operating both in research processes and in diagnostic practices within clinical settings (Bacigalupe et al., 2024; Walther, 2025). In particular, dominant theoretical frameworks and commonly used assessment instruments tend to operationalize psychological distress primarily through internalizing manifestations (e.g., sadness, crying, guilt, or anxiety) (Bacigalupe et al., 2024; Cavanagh et al., 2017), which may facilitate identification when distress is expressed in these terms while simultaneously under-detecting more externalizing or non-normative presentations in men (e.g., irritability, risk-taking behaviors, substance misuse, or aggression) (Sedlinská et al., 2021; Streb et al., 2021). In this sense, bias in evidence production and diagnostic routines may contribute to an underestimation of the prevalence of affective or anxiety disorders among men and to the reinforcement of observed differences that, at least in part, reflect methodological and clinical limitations rather than true variation in the burden of distress.

In the same line, Camacho-Ruiz et al. (2024) pointed out that men usually display more barriers when it comes to asking for help. These barriers are mainly related to the social stigma associated with showing vulnerability, which is linked to weakness or femininity. Other barriers include lack of knowledge and difficulties establishing a therapeutic bond with mental health professionals. Consequently, men may not access care resources or, when they do attend, they may present in a more deteriorated condition (Camacho-Ruiz et al., 2024). This directly affects men’s therapeutic adherence (Addis & Mahalik, 2003; Levant et al., 2013). These findings also suggest that difficulties in help-seeking, associated with barriers to emotional expression related to vulnerability, are grounded in gender-differentiated socialization processes in which traditional masculinity values are predominant, such as self-sufficiency, control, and rationality over emotionality (Camacho-Ruiz et al., 2024; Galdas et al., 2005; Mahalik et al., 2003; Real, 2003).

Therefore, we are confronted with patriarchy as a historically forged system that has shaped men’s relationship with mental health and associated treatments. These treatments or therapeutic models suggest that men adopt behaviors that favor strength and emotional toughness and, therefore, create barriers to emotional suffering while also hindering the establishment of a therapeutic bond that fosters the trust necessary for emotional expression and a more subjective and personalized intervention (Englar-Carlson & Kiselica, 2013; Gough, 2016; Gough & Robertson, 2010).

### 1.2. Gender-Responsive and Intersectional Approaches to Men’s Mental Health

In general, men have not been recipients of interventions with a gender perspective. It is necessary to start from this point, since this perspective shows how different ways of living masculinity interact across distinct social and cultural contexts. For that reason, it is necessary to maintain an intersectional perspective, since it is necessary to consider the different variables that interact in different forms of oppression, as well as in different privileges. That is, it is necessary to understand the different dimensions that can influence a therapeutic process for many men; for example, ease of access to treatment is not the same for a man from a wealthy neighborhood as for a man from a disadvantaged neighborhood. It is necessary to take this dimension of social class into account, as well as age, ethnicity, sexual orientation, gender, or other comorbid pathologies (Camacho-Ruiz et al., 2024; Crenshaw, 1989; Frosh et al., 2017; Hooks, 2021). For this reason, feminist theories have paved the way towards understanding that masculinity cannot be analyzed in the singular, that not all mental health problems presented by men are the same, and that it is necessary to begin to understand intervention from subjectivity and intersectionality (Crenshaw, 1989), as well as to recognize that the construction of masculine identity obeys gender norms generated through processes of differentiated socialization.

For this reason, it must be considered that existing care resources, although numerous advances have been developed in interventions and methodologies relevant to men, usually still maintain androcentric or neutral perspectives. Intervention with a gender perspective in men, which considers the influence of gender norms of differentiated socialization, such as “*being tough*” or “*being strong*”, or other norms that may affect barriers or resistance to accessibility or bonding with therapeutic processes, continues to be limited and reduced in many clinical contexts. On purpose, authors such as Jordan et al. (2012) or Messner (2016) highlight the scarcity of introducing the gender perspective and the limited training that mental health professionals receive in this regard. This results in the absence of specific therapeutic instruments to intervene and counteract the costs of hegemonic masculinity, which promote strength and self-sufficiency as fundamental values to be a “*good man*”.

If these types of interventions are generated and strategies and therapeutic instruments are created, a critical and novel perspective is offered in the analysis of men’s psychological suffering, and it would intervene on the subjective barriers and resistances that reduce men’s access to mental health resources. Different studies indicate the presence of patterns that are repeated. Younger men more consistently reproduce patterns in which, as a tendency, emotional problems are minimized by avoiding being seen crying and by avoiding expressing fear and pain, as well as by reducing behaviors or actions that reveal emotional vulnerability (Camacho-Ruiz et al., 2024; Frosh et al., 2017). Within these patterns, help-seeking, a determinant of health, is perceived as a threat to a masculinity that is validated through identification with the peer group (Robertson et al., 2016; Seidler et al., 2016). In this construction of masculine identity through peer groups, vulnerability is viewed as weakness and asking for help as the opposite of “*being a real man*”. The consequences, on many occasions for many men, include the prolongation of suffering and emotional distress. This way of silencing discomfort often leads to problems related to addictions, violence, and isolation (Camacho-Ruiz et al., 2024; Hooks, 2021).

Authors emphasize that many men adopt thoughts, emotions, and values in which they take for granted that psychological suffering must be endured alone and, in more extreme cases, denied and rejected. Therefore, when some men find themselves in a critical situation, the motivation to ask for help and the accompaniment to resources or to mental health specialists comes through their closest environment. This set of actions or model is usually called the “*last resort*” model (Addis & Mahalik, 2003; Vogel et al., 2011). These actions, influenced by behaviors associated with procrastination, are intensified by mental health systems that have not incorporated a transversal gender vision in their care devices, which frequently generates a feeling of disidentification among most men with mental health care resources and therapeutic spaces.

Although these barriers persist, a degree of optimism is warranted. Promising responses and alternatives to traditional treatments are beginning to emerge. Some professionals incorporate new psychotherapeutic methodologies, such as compassion focused therapy, acceptance and commitment therapy, or more current models that consider how hegemonic masculinity negatively impacts the socialization processes of many men. These more current clinical models highlight new ways for men to relate to distress (Addis & Mahalik, 2003; Mahalik & Rochlen, 2006; Seidler et al., 2016; Wong & Wester, 2016). These interventions seek to place the focus on the legitimation of emotional experience, to respectfully question traditional models of masculinity, and to explore new paths in subjective elaborations and identity transformations.

In addition, the need to generate new qualitative research that helps to understand the existing relationship between men and illnesses related to mental health must be considered. Indeed, these investigations are considered entirely necessary to understand the complex dynamics that exist in the relationship between men and their mental health and the influence of stigmas generated through gender-differentiated socialization processes. In the context of mental health research, using in-depth interviews and discussion groups is especially relevant, as these approaches often yield data that cannot be obtained through quantitative methodologies. These accounts will help to create specific interventions with a gender perspective, focusing on exploring the difficulties that some men present when asking for help, on how men perceive symptoms, and on analyzing the effects of masculinity on the evolution of their emotional problems (Camacho-Ruiz et al., 2024; Oliffe et al., 2022; Rice et al., 2018b; Seidler et al., 2018, 2020).

However, the analysis of masculinity in mental health interventions with a gender approach in men should not only be carried out with men who need treatment. In addition, it is important to reflect on the epistemological and clinical foundations through which psychiatry and psychology approach interventions with men, and to consider whether gender-related assumptions may influence assessment and treatment in certain settings (Bailey et al., 2020; Merone et al., 2022; Verdonk et al., 2009). It is necessary to consider that the majority of disciplines and fields of study have historically been created through an androcentric vision, which has normalized men’s problems as neutral (Bailey et al., 2020; Merone et al., 2022; Verdonk et al., 2009). By introducing the gender perspective, an opportunity is offered to move towards a clinic based on an exercise of epistemic and social justice, supported by an ethical and moral responsibility.

### 1.3. Study Objective

Based on previous findings, this research arises from the need to deepen understanding in this emerging field of reflections and practices in mental health intervention with men. Its main objective is to examine how hegemonic masculinity norms and gendered socialization processes shape attitudinal and structural barriers to men’s access to mental health resources, and to outline gender- and intersectionality-informed therapeutic strategies to enhance treatment engagement, adherence, and outcomes. For this, it is necessary to combine studies of critical masculinities, current psychotherapeutic models, an intersectional perspective that considers the plurality of masculinities, and feminist theories, thereby generating an integrative approach. The proposal is clear: to understand the complexity of silenced male suffering and to identify psychotherapeutic intervention strategies that are more inclusive and that result in more ethical and, above all, effective actions.

What is intended is to awaken concerns when building new clinical practices for men who demand assistance in mental health resources, all of them based on different ways of moving in a healthier way through subjective self-knowledge of masculinity, and that support an identity construction that emphasizes care, vulnerability, and a more diverse emotional expression, without gender biases.

This research is also aligned with the United Nations Sustainable Development Goals (SDGs), particularly SDG 3 (Good Health and Well-being) and SDG 5 (Gender Equality), by addressing gender-related determinants that shape access to and engagement with mental health care and by seeking to reduce avoidable disparities in prevention, diagnosis, treatment initiation, and follows-up. By synthesizing evidence on masculinities, help-seeking barriers, and gender-sensitive therapeutic models, the current review provides clinically actionable knowledge for designing interventions that are more responsive to men’s socialized patterns of distress and communication, strengthening the therapeutic alliance, improving adherence, and facilitating earlier and more effective care. In this sense, the study reinforces the relevance of integrating gender and intersectionality as cross-cutting clinical variables, supporting ethical and evidence-informed practice aimed at expanding access, enhancing quality of care, and promoting more equitable mental health outcomes.

## 2. Materials and Methods

This study adopts an exploratory narrative review design, with the objective of examining how hegemonic masculinity norms and gendered socialization processes shape attitudinal and structural barriers to men’s access to mental health resources and engagement with care, and of outlining gender- and intersectionality-informed therapeutic strategies to enhance treatment adherence and outcomes. It is an integrative analysis that considers both qualitative and quantitative studies, with special attention to research that explores masculine constructions of distress, barriers to professional help seeking, and gender sensitive therapeutic models. The general purpose of the current narrative review was to map the current state of knowledge, identify research and clinical gaps, and collect relevant contributions for clinical practice with men from a non-androcentric perspective. The search strategy was designed to capture the most recent and most significant literature in the field. The search was conducted in three academic databases of high relevance in the social sciences and health: PubMed, Scopus, and Web of Science (WOS). Both controlled descriptors (Medical Subject Headings—MeSH) and free-text search terms were used, combined through Boolean operators to ensure broad and precise thematic coverage. The terms included: “men’s mental health”, “masculinities”, “gender and psychotherapy”, “help-seeking behavior in men”, “gender-sensitive interventions”, “hegemonic masculinity and mental health”, among other equivalent terms in English and Spanish.

The inclusion period was limited to 2015–2025 in order to include recent and relevant contributions in a field that is constantly evolving. Filters were applied to restrict results to peer-reviewed articles and published in English or Spanish. In addition, the bibliographies of the selected studies were manually reviewed to identify complementary works not detected in the initial search. The included studies had to meet the following criteria: (a) focusing on adult men (18 years or older); (b) addressing topics linked to mental health, therapeutic intervention, and/or psychological distress; (c) using qualitative, quantitative, or mixed methodologies; and (d) having been published between 2015 and 2025, in English or Spanish.

Studies were excluded if they (a) were not original research (for example, editorials, letters to the editor, reviews without critical analysis) or (b) were duplicated studies. Consistent with narrative review methodology, we did not conduct a formal risk-of-bias assessment; however, study characteristics were extracted systematically and findings were interpreted with attention to methodological heterogeneity. For data analysis, findings from the included studies were organized into thematic categories using descriptive and comparative techniques to identify patterns across the evidence base. Data synthesis followed a narrative, interpretive approach. The three thematic dimensions served as a conceptually informed organizing framework, grounded in the literature and iteratively refined through close reading and comparison of the included studies. This approach aimed to integrate recurring patterns and explanatory themes across studies, rather than to conduct a formal inductive thematic synthesis.

## 3. Results

In total, eleven (Boettcher et al., 2019; Edwards et al., 2017; Kwon et al., 2023; Lynch et al., 2018; Mesler et al., 2022; Mostoller & Mickelson, 2024; Seidler et al., 2021a; Sharp et al., 2023; Stewart & Harmon, 2004; Tibubos et al., 2022; Wilson et al., 2022) empirical studies were analyzed, involving a total of 7175 participants. These studies employed both qualitative and quantitative methodologies, with the majority of included studies being quantitative. Table 1 provides an overview of the studies included in the narrative synthesis, detailing methodology, country/setting, sample characteristics (including gender and age), and additional diversity indicators when available from the original reports.

**Figure 1:**
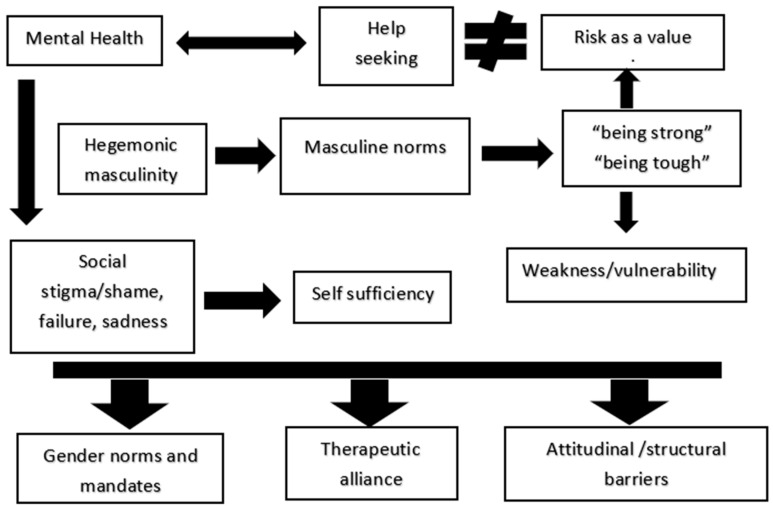
Main Factors associated with Mental Health in Men. **Notes:** This framework reflects an interpretive, narrative synthesis of key themes and proposed linkages identified across the literature. Single-headed arrows denote relationships typically presented as directional; double-headed arrows denote bidirectional relationships. Where directionality was unclear or not explicitly stated, connections are presented as conceptual associations. Different from (or distinct from) is denoted by ≠. Risk as a Value: Risk-taking is culturally framed as a desirable, status-enhancing marker of masculine credibility (e.g., toughness and self-mastery), whereas caution or help-seeking may be viewed as weakness. The figure summarizes a conceptual model and does not imply causal inference. The figure was developed by the authors based on a narrative integration of recurring themes and relationships identified in the literature.

To facilitate interpretation of the results, they are analyzed and organized into three dimensions (see Figure 1 for further details). The first one concerns how traditional masculine norms—such as emotional repression, self-reliance, and the central mandate of hegemonic masculinity (e.g., “*being strong*”)—hinder a fundamental action for mental health, namely help-seeking. It is associated with a message that many men hear throughout their lives is “*men do not cry*”. It is necessary to analyze how hegemonic masculinity oppresses women and represses men (Hooks, 2021). Perhaps it is increasingly less present within the family, but it remains highly present within peer groups during adolescent development. An illustrative example concerns the emotion of fear: this emotion should not be shown, since, within hegemonic masculinity and particularly in adolescence, risk is often construed as a value (i.e., socially valued risk-taking linked to hegemonic masculinity, including physical, social, and health-related risk). Risk as a value refers to the normative cultural framing of risk-taking as inherently desirable and status-enhancing: an indicator of competence, courage, and masculine credibility. Within hegemonic masculinity, willingness to incur physical, social, or psychological risk is often positioned as proof of toughness and self-mastery, while caution, help-seeking, or avoidance may be coded as weakness or lack of control. As a result, risk-taking becomes not only a behavior but a moralized standard through which men’s identities are judged and policed in peer and institutional contexts.

Therefore, men who feel fear are perceived as weak or vulnerable. Self-reliance, or solving problems by oneself, is a behavior closely related to self-sufficiency, which often delays men from asking for help in difficult moments. Within hegemonic masculinity, help-seeking portrays a man who is not capable of providing a solution to his problems by himself, and thus as a weak man, contrary to the main norms or mandates of hegemonic masculinity, such as “*being strong*” or “*being tough*”.

Therefore, help-seeking contradicts what it means to be “*a real man*”. Firstly, seeking help signals a lack of strength and, therefore, being less of a man. Second, both attitudinal (personal) barriers and structural barriers are identified. Personal barriers include traditional masculinity norms that have already been adopted by many men and have become part of the construction of their identity. That is, many men have incorporated into their belief systems and cognitive structures the idea that they must be strong and self-sufficient, generating self-stigma if they perceive that they do not comply with internalized mandates and may ultimately be seen as “*not very manly*”. In these cases, other emotions linked to help-seeking may emerge, such as shame or a sense of failure, creating stigmas that can lead to anguish, anxiety, or sadness. In these contexts, help-seeking is also associated with an inability to take care of oneself, because men must maintain an image of self-sufficiency: “*I can do it alone*”. Structural barriers include lack of information about available resources, lack of resources offering interventions specifically for men from a gender perspective, and the costs of treatment, which marginalize men who lack the means to access psychological care. Finally, it is important to emphasize the lack of bonding or connection with mental health professionals and the perception that therapy is not useful. These factors are the main causes of men’s discontinuation of mental health services identified by studies analyzed.

In the final section of the results, the main limitations of the studies included in this review are examined, highlighting methodological and contextual constraints that may affect the interpretation and transferability of the findings.

### 3.1. Traditional Masculinity Norms as Barriers to Men’s Mental Health Help-Seeking

The mandates and norms of hegemonic masculinity—such as the belief that a man must be capable of confronting and solving his problems by himself, emotional repression, and strength as the central mandate of hegemonic masculinity—constitute the main characteristics of masculinity that lead men to avoid seeking help when facing mental health problems. These norms or mandates, deeply rooted in masculine socialization, reinforce the idea that expressing emotions or requesting professional help represents a sign of weakness, thereby threatening masculine identity and belonging to the peer group governed by these masculinity norms. Consequently, many men tend to avoid professional help in order to protect their personal and public image, resorting to different coping mechanisms, such as silence or the consumption of alcohol or other drugs (Lynch et al., 2018; Wilson et al., 2022).

Aligning with these gender mandates, which emphasize independence, invulnerability or the absence of weakness, and courage, generates psychological barriers that make it difficult to recognize psychological distress and to seek help. For Kwon et al. (2023), men perceive that help-seeking threatens their masculine identity, which consequently leads them to avoid discussing issues related to mental health or self-care. This phenomenon is reinforced by the social stigma associated with vulnerability and by negative experiences such as criticism or mockery in social contexts, including peer groups across different spaces of public life—from friends to workplaces or social networks—contributing to the minimization of mental health problems, delays in help-seeking, and a preference for more dangerous coping strategies, such as substance abuse.

In addition, it has been indicated that these mandates, centered on the rejection of vulnerability and influenced by aggressiveness and emotional control, enter into direct conflict with the requirements of therapy, which involve expressing emotions related to care (leaving one’s comfort zone), admitting failures, and yielding control in an egalitarian way. These contradictions produce shame and resistance to therapeutic treatment, which helps to explain the high levels of risk behaviors, substance abuse, violence, and suicide observed in men (Stewart & Harmon, 2004).

For Boettcher et al. (2019), three dimensions through which these norms operate are identified: (I) injunctive norms, which dictate what “*should*” be done and promote the avoidance of vulnerability; (II) descriptive norms, which are transmitted through the observation of other men and reinforce perseverance in the face of stress; and (III) cohesive norms, shaped by male leaders who reward resistance and self-sufficiency. Therefore, it must be considered that these ways of living masculinity reinforce the idea that help-seeking is incompatible with what hegemonic masculinity understands as “*being a man*”, especially in social and cultural environments in which men predominantly engage in different activities.

According to Edwards et al. (2017), although some studies do not show these norms directly, it has been observed that male caregivers are significantly more likely to report limited social or emotional support (Edwards et al., 2017). This finding suggests that the lack of support networks may be mediated by norms that make both the expression of vulnerability and help-seeking more difficult.

Regarding the therapeutic space, some men who identify with these norms or mandates tend to experience shame and resistance when initiating therapeutic processes, showing less optimism about their usefulness and less commitment to the process. On some occasions, this type of behavior contributes to higher dropout rates and creates distance, or makes it difficult to establish rapport, with mental health professionals (Seidler et al., 2021a). Additionally, Seidler et al. (2021a) note that men who avoid treatment tend to believe that they must solve their problems by themselves, generating mistrust regarding treatment outcomes. The main consequence is the formation of attitudinal barriers that are intrinsically related to the values of hegemonic masculinity and represent an important obstacle for the treatment.

This type of masculinity and its gender norms is also related to other risk behaviors in health and in social and cultural dimensions. In the study by Tibubos et al. (2022), it is pointed out that hegemonic masculinity is, on some occasions, characterized by maintaining traits that orient aggressive and power-related behaviors, promoting stigma toward behaviors and thoughts that can be considered related to emotional vulnerability, thereby generating barriers to accessing psychotherapeutic treatments.

Moreover, in a study conducted by Sharp et al. (2023), a specific cultural context was analyzed: Australia. In this study, the norms of hegemonic masculinity maintain a strong influence in terms of their assimilation by many men. For these men, these norms are acquired from very early ages, mainly self-sufficiency and emotional toughness, often leaving space for the expression of anger, while emotions more closely related to care activities such as tenderness, affection, or sadness are repressed. The persistence of these highly masculinized emotional patterns primarily generates emotional disconnection and isolation, which often results in unhealthy resolutions of mental health-related problems, such as substance use. More optimistically, it is also evidenced that some men have begun to question and challenge these norms.

In addition, Mesler et al. (2022) and Mostoller and Mickelson (2024) argue that many men experience constant pressure to comply with standards associated with traditional or hegemonic masculinity, generating psychological distress due to perceived discrepancies between complying with these gender norms and maintaining emotional balance, which increases the likelihood of experiencing higher levels of depression and anxiety. The acceptance of these norms is directly associated with stigmas related to help-seeking, creating a norm of solving problems by oneself, and a performativity based on heteronormativity and the image of power over women as fundamental characteristics of self-concept and self-image.

### 3.2. Hegemonic Masculinity and Structural Constraints Limiting Men’s Access to Mental Health Resources

Hegemonic masculinity and structural constraints limit men’s access to mental health resources. In this section, we outline the barriers identified, which are primarily attitudinal and structural in nature. These barriers exert a strong influence on the main problems in access to mental health resources among many men.

Regarding attitudinal barriers, many men feel that they must solve their problems by themselves. As has already been discussed throughout the text, this norm is associated with a specific way of masculinity in which other norms such as strength, toughness, self-sufficiency, and self-reliance are exalted (Lynch et al., 2018; Stewart & Harmon, 2004; Wilson et al., 2022). This way of living masculinity, which emphasizes rationality, control, and independence, has as its main consequences that help-seeking is perceived as a sign of vulnerability and therefore of weakness or dependence, and that symptoms of possible mental health problems are minimized. These consequences result in resistance to seeking psychotherapeutic care resources or, when help is sought, in presentations where the problem may have worsened.

It must also be considered that these attitudinal barriers are generated through processes of differentiated socialization by gender issues. This type of masculine socialization implies the internalization of these norms by men, generating values and thoughts associated with maintaining control and managing problems with total independence (Seidler et al., 2022). Behaviors may emerge in which many men prematurely interrupt their therapeutic process or self-evaluate, mostly without professional guidance and in an erroneous way. In addition, attending therapy is, on many occasions, contradictory to what has been learned through socialization processes, since it may imply a loss of emotional control and of power and authority. Therefore, attending therapy can generate reactivity due to the factors described above, which may also hinder treatment engagement.

In addition to gender-specific frameworks, the findings can also be interpreted through Andersen’s Behavioral Model of Health Services Use, which conceptualizes help-seeking as the result of predisposing factors, enabling resources, and perceived need (Andersen, 1995). From this perspective, masculine norms operate as predisposing factors that shape attitudes toward vulnerability and self-reliance, consistent with the framework proposed by Addis and Mahalik regarding men’s help-seeking (Addis & Mahalik, 2003). Likewise, structural characteristics of mental health services may function as enabling or constraining conditions. Taken together, these models provide a useful multilevel lens for understanding how gender norms interact with contextual and systemic factors in shaping men’s help-seeking behaviors.

On the other hand, structural barriers include limited knowledge about available services for early care. In addition, the difficulty that many men encounter, on many occasions, in navigating the health system, as well as the economic costs associated with treatment, are often barriers that are difficult to overcome (Kwon et al., 2023; Seidler et al., 2022; Seidler et al., 2021a). It is also essential to consider an intersectional approach when analyzing mental health in men, since factors such as age, social class, ethnicity, or culture are particularly relevant to men’s access to mental health care. Economic vulnerability therefore becomes a fundamental barrier that prevents men from accessing treatment or, in many cases, leads them to discontinue care before completing the therapeutic process.

Additional problems related to structural barriers include delays in care, bureaucracy, and the repetition, in many cases, of personal experiences, which may become traumatic, within the health system and with different professionals. This, combined with processes that already generate shame due to socialization, may produce demotivation and its consequent abandonment of the process or of help-seeking (Kwon et al., 2023; Seidler et al., 2022; Seidler et al., 2021a). In addition, it must be added that there is a lack of awareness campaigns that promote the reduction in stigma when men talk about mental health. Prevention in this area, and from a gender perspective, is fundamental for advancing toward more effective treatments and earlier assessments.

Stewart and Harmon (2004) highlight that structural barriers also reinforce the stigma linking masculinity with self-sufficiency, perpetuating the exclusion of men from mental health services and contributing to their representation as “*difficult to treat*”. Boettcher et al. (2019) add that these limitations, both internal and external, are intensified in work environments where expectations of performance and strength reinforce the idea that requesting help is incompatible with masculinity.

In relation to this, Edwards et al. (2017) found that men who participate in care-related activities report better access to social and emotional support, which indicates that gender-socialization processes generate structural and attitudinal barriers that deny access to both informal and formal networks of help. That is, through processes of differentiated gender socialization, it is internalized that care activities, within hegemonic masculinity, are not “*men’s things*”. This can be clearly observed in the games that boys and girls engage in during socialization. Boys’ games have traditionally been oriented toward adventure and action, whereas girls’ games have mainly been oriented toward toys and activities related to care. Therefore, this dependence on partners or family members as fundamental sources of support shows how gender influences professional help-seeking, in which, many times, those closest to men motivate them to seek help when they can no longer cope.

Following this line, the study by Tibubos et al. (2022) analyzes how gender norms and mandates learned through socialization foster self-reliance and reject emotional vulnerability in men and, moreover, are fundamental to the persistence of these barriers. They also contribute to the creation of stigmas that portray men as weak when they seek help. For Sharp et al. (2023), in settings such as Australia, values related to emotional repression and independence further strengthen this image. If help is ultimately sought, for many men it is a sign of personal failure, which increases isolation and as indicated above, significantly reduces opportunities for early care, thereby increasing the likelihood that the problem will worsen.

Emphasizing an intersectional perspective, the research by Mesler et al. (2022) shows that in some cultural and social contexts in which gender norms and mandates of hegemonic masculinity are more strongly internalized, stigmas linked to seeking psychological treatment are further reinforced. In turn, the study by Mostoller and Mickelson (2024) highlights the interaction between attitudinal and structural barriers as a joint system that limits access to care. In fact, the perception of dependence on others, associated with beliefs about weakness or vulnerability, must be considered together with barriers such as treatment costs or limited knowledge of resources, which foster emotional distress and the non-use of mental health services by many men.

### 3.3. Weak Therapeutic Alliance and Perceived Ineffectiveness as Drivers of Treatment Dropout Among Men

On many occasions, the perception that therapy is not useful and the lack of connection with mental health professionals are two of the most frequent reasons why men discontinue mental health services, according to the studies reviewed (Boettcher et al., 2019; Lynch et al., 2018; Wilson et al., 2022). These two issues are closely related to traditional masculine norms, which discourage emotional expression, vulnerability, and the seeking of external support, thereby often limiting the development of strong therapeutic relationships.

According to Kwon et al. (2023), there is considerable convergence across numerous studies regarding what many men experience in therapy in relation to the impersonality of therapeutic processes, in which they perceive that professionals do not show genuine interest or do not understand their experiences in a contextualized manner. This sense of mistrust and lack of empathy leads to demotivation and, in many cases, to premature treatment dropout. In addition, in some instances, the lack of transparency in the formulation of therapeutic goals and the absence of a results-oriented approach reinforce the perception that therapy does not meet their expectations or provide practical solutions to their problems. The absence of a gender perspective means that objectives related to the costs of hegemonic masculinity are not considered, particularly those associated with emotional repression and the rejection of vulnerability, as well as the difficulty in establishing affective bonds with other men.

The findings of Seidler et al. (2021a) are particularly illustrative: 54.9% of men reported having discontinued therapy due to a lack of connection with the therapist, while 20.2% indicated that they considered it of little use or ineffective. These perceptions reflect both the persistence of attitudinal barriers and the need to adapt interventions to the specific characteristics of men, considering their expectations, communication styles, and conceptions of help. In fact, Seidler et al. (2021a) found that men who rejected therapy were significantly more likely to believe that psychotherapists would not understand their problems or that psychotherapy would not be effective, demonstrating clear mistrust in the usefulness of professional treatment (Seidler et al., 2022).

Therefore, this disconnection may originate from the persistence of therapeutic approaches that do not incorporate a gender perspective in a cross-cutting manner and thus fail to recognize men’s specific needs or their preference for more pragmatic strategies primarily oriented toward action and rationality. In fact, when men do not obtain tangible and immediate results, they assume that therapy is ineffective and consider solving their problems on their own. These responses reinforce self-sufficiency and frame help-seeking as negative (Boettcher et al., 2019; Mesler et al., 2022).

Moreover, the perception that therapy is not useful is reinforced by previous negative experiences and by the belief that psychological intervention will not address underlying problems, generating an attitude of skepticism toward mental health services (Lynch et al., 2018). In contexts where traditional gender norms are more rigid, this skepticism intensifies, as expressing emotional difficulties or relying on professional help is perceived as a threat to masculine identity (Tibubos et al., 2022).

In the study by Mostoller and Mickelson (2024), it is emphasized that men who adhere to hegemonic masculinity values that promote self-sufficiency, emotional control, and rationality maintain that therapy is incompatible with their needs, which increases dropout from therapeutic processes. These authors stress the need to implement gender-sensitive interventions that emphasize closer and more empathic communication, are grounded in an ethics of care, and include clear goals that foster increased trust and therapeutic progress.

In another study, participants indicated that seeking professional help may generate negative social repercussions, such as labeling, adverse reactions, perceptions of weakness, and possible rejection by the group. This occurs because peer-group values often regard help-seeking as a sign of weakness, which discourages men, especially young men, from seeking professional support. In addition, all participants reported that self-medication with alcohol is the most prevalent and socially accepted method for coping with difficult emotions. Alcohol is used as a means of escape and to numb emotional problems. This behavior reflects a deeper cultural issue related to the acceptance of alcohol as a coping strategy (Lynch et al., 2018).

In another study by Wilson et al. (2022), many participants also highlighted the relevance of having male role models who convey an alternative form of masculinity that promotes care-related values. They believed it is necessary to have educational experiences that help individuals build their identity by reflecting on other, more egalitarian and positive masculine models. They considered it essential to break with traditional stigmas that normalize behaviors restricting emotional expression and help-seeking. They also emphasized that contact with such role models can be crucial for many men, including young men, to develop a healthier masculinity. Furthermore, they concluded that it would be highly important for these types of initiatives to be incorporated transversally into curricular activities throughout the entire educational process of all children and adolescents.

### 3.4. Study Limitations of the Analyzed Studies

The main limitations identified in the studies analyzed refer to the predominance of studies conducted in English-speaking countries, mainly Australia, England, and Canada. This may restrict the analysis of data obtained from other sociocultural realities and may therefore limit a more intersectional understanding of the phenomenon under study. It would be valuable to incorporate data from countries with different sociocultural contexts, such as Southern European countries, Latin America, or Asia.

Another aspect that should be highlighted, and that also contributes to the limited intersectional approach, is that the majority of studies has focused on heterosexual men. This excludes other experiences of living masculinity, such as those of LGTBIQ+ individuals and non-binary people, which Connell refers to as subordinate masculinity (Connell & Messerschmidt, 2005). These experiences and lived realities may reveal other patterns or complexities derived from hegemonic masculinity norms and their relationship with mental health.

Finally, continuing to emphasize an intersectional approach, and also due to the difficulty of finding studies addressing these variables, the experiences of older men have not been analyzed in deep, as the literature identified and reviewed refers mainly to men in middle adulthood and adolescence. This precludes comparison with men who developed their socialization under more traditional masculine mandates, which would have helped to clarify the impact of gender on mental health across the life course of many men. In addition, there is no reference to men in situations of extreme vulnerability, which Connell refers to as marginalized masculinity (Connell & Messerschmidt, 2005). This also contributes to the scarcity of data on profiles whose main characteristic is limited economic means to access mental health treatment.

## 4. Discussion

The review conducted reveals certain structural and subjective dimensions that interact with, or maintain a relationship between, gender, masculinities, and mental health. Cultural and religious contexts can meaningfully shape how masculinity is constructed and performed, with downstream implications for men’s mental health and patterns of formal help-seeking. In the WHO Regional Office for Europe region, evidence suggests that widely valorized masculine norms (e.g., self-reliance, emotional restraint, and self-control) may function as sociocultural barriers to recognizing distress and seeking professional support, although their expression and salience vary across settings (Gough & Novikova, 2020). In some Hispanic/Latinx contexts, machismo has been described as a salient normative frame that can discourage the disclosure of vulnerability and reduce willingness to access mental health care (Espí Forcén et al., 2023). Religious traditions may also influence help-seeking through gendered moral expectations and community norms: for example, work with Evangelical Christian samples indicates that certain religious beliefs and orientations are associated with attitudes toward psychotherapeutic engagement (Lloyd et al., 2021). Similarly, research on Arab-Muslim communities highlights how stigma, culturally inflected explanatory models of distress, and preferences for religious/community-based sources of support may shape pathways into (or away from) formal mental health services (Alhomaizi et al., 2018).

Addis and Mahalik (2003) point out that hegemonic masculinity and its gender norms or mandates continue to constitute a barrier to access, continuity, and the effectiveness of treatment for men with mental health problems. When a gender perspective is incorporated into research, findings indicate the existence of common patterns that help shape a form of masculine socialization based on clear norms of emotional repression and self-sufficiency, and on the understanding of vulnerability as equivalent to weakness, leading to the rejection of behaviors or emotional expressions associated with vulnerability (Hooks, 2021). These patterns coincide with the core characteristics of hegemonic masculinity proposed by Connell (Connell & Messerschmidt, 2005).

The hegemonic masculinity formulated by Connell and Messerschmidt (2005) not only constructs a normative and performative ideal that generates positions within gender relations between men and women but is also responsible for imposing discipline on the ways of expressing, experiencing, and navigating psychic suffering. This also influences how bonds are established, how men communicate and seek help, and, fundamentally, how emotional suffering is symbolized, especially within peer groups (Courtenay, 2000; Kimmel, 2019).

The reviewed studies by Gough (2006) and O’Neil (2008) indicate that difficulties in help-seeking are not individual or pathological issues but rather respond to cultural and social norms learned through socialization processes. For this reason, the minimization of distress, the non-expression of emotions, and resistance to treatment can only be understood within a symbolic framework that represses men’s emotional expression. The existing literature further shows that both attitudinal and structural barriers operate independently and are reinforced by gender mandates, which prolongs the low rate of help-seeking for mental health problems among men (Boettcher et al., 2019; Edwards et al., 2017; Kwon et al., 2023; Lynch et al., 2018; Mesler et al., 2022; Mostoller & Mickelson, 2024; Seidler et al., 2021a; Sharp et al., 2023; Stewart & Harmon, 2004; Tibubos et al., 2022; Wilson et al., 2022), and indicates the need for strategies to comprehensively address men’s difficulties in accessing mental health care, considering cultural, psychological, and practical variables.

Taking this into account, some men may conceal their suffering related to the lack of informal care; even in highly distressing circumstances, the norm of invulnerability can persist, while simultaneously highlighting potential limitations of traditional masculinity when emotional openness is required (Courtenay, 2000; Oliffe et al., 2011).

It is also important to highlight that the evidence extracted from the studies analyzed in the review brings to light the effects of hegemonic masculinity that remain present today in how many men manage, express, and cope with psychological suffering. Throughout the research, it has been demonstrated that it is not only necessary to consider the lack of affective skills among some men, but it is also essential to emphasize how, at a cultural and social level, the idea that vulnerability is a sign of weakness and that a lack of self-sufficiency represents a clear loss of prestige for many men within their peer groups is deeply rooted. Taking all of this into account, an action that is decisive for health, such as help-seeking, becomes a clear threat to masculine identity. This results in the naturalization of emotional silence when facing psychological suffering. However, behind this apparent masculine strength, there is an emotional isolation that amplifies psychological distress and hinders many men’s access to therapeutic support resources (Seidler et al., 2022). In summary, norms that emphasize the rejection of vulnerability in men—particularly those related to “*being strong*,” “*being tough*,” or “*I can do it alone*”—are not only an issue to be considered in mental health-related pathologies; they also directly influence the establishment of healthy and authentic affective bonds. This highlights the relevance of developing models of masculine socialization that prioritize care and the acceptance of vulnerability as something human and necessary when relating to others.

In the study by Camacho-Ruiz et al. (2024) evidence indicates that men experience greater difficulties than women when seeking help for addiction problems and, furthermore, that once they do seek help, they present with more advanced deterioration, which, on many occasions, has consequences for their recovery, as well as for treatment adherence and the outcomes of therapeutic intervention (Addis & Mahalik, 2003; Levant et al., 2013). In line with these conclusions, the results highlight the need to provide gender training to professionals so that they maintain a gender-sensitive approach, promoting interventions and criteria that help overcome problems derived from barriers associated with stigmas produced by hegemonic masculinity, which keep many men experiencing difficulties when seeking help in order to receive therapy in the area of mental health. This gender training is also connected to an important structural barrier, since many mental health resources remain conditioned by ways of operating based on androcentric or neutral logics, which do not consider how gender norms influence demand, resistance, and symptomatology among men who need to be attended to for problems derived from mental health (Bailey et al., 2020; Merone et al., 2022; Verdonk et al., 2009).

The evidence provided by the studies (Boettcher et al., 2019; Lynch et al., 2018; Mesler et al., 2022; Mostoller & Mickelson, 2024; Tibubos et al., 2022; Wilson et al., 2022) indicates that the lack of a therapeutic bond and, on many occasions, the perception of inefficacy among many men do not have their roots solely in individual factors; sociocultural dynamics also influence the ways in which men seek psychological help. Therefore, changes are required in clinical practices that responsibly assume a gender-sensitive therapeutic perspective when working with men, offering a safe and collaborative space in which they can express their vulnerability, within a sexuality that is not marked by the values of hegemonic masculinity in which virility, initiative, constant availability, and heteronormativity prevail as fundamental values, within egalitarian everyday relationships with others, in their affective relationships with people close to them, including other men, and also based on an ethics of care and co-responsibility, considering that all of this does not constitute a threat to their identity as men. Therefore, if the findings indicate that hegemonic masculine norms hinder early access or non-access to care (Connell & Messerschmidt, 2005; Hooks, 2021; Seidler et al., 2022), and also influence the quality of the therapeutic alliance, treatment adherence, and the identification of psychological distress, it is necessary that masculinity should become a clinical variable to be taken into account transversally in any process of psychological intervention with men.

Therefore, as previously emphasized, when a man initiates a therapeutic process, research indicates that increasingly specific dynamics that must be taken into account by professional teams become evident, including the complexity of verbalizing complex emotional states, difficulties in discussing issues related to sexuality, distrust toward an ethics of care, or the tendency to resort to technical or rational discourse as a defense against introspection (Englar-Carlson & Kiselica, 2013; Gough, 2006; Seidler et al., 2021b). Therefore, specialized forms of care must be proposed that do not focus solely on subjective resistance to change, but that understand these resistances as the product of gender-differentiated socialization operating at a structural level, and as causes of identities or subjectivities that shape the ways in which many men reject vulnerability because it is viewed as weakness and therefore contradicts the core norm of hegemonic masculinity: “*being strong*”. It is essential to understand that within hegemonic masculinity, emotional repression has been viewed as necessary and even positive, since traditionally men’s life projects were marked by an ideology in which they acted as providers and family protectors. Therefore, this, often forced men to internalize mandates related to toughness and strength, extrapolating these mandates to their affective and care-related domains (Connell & Messerschmidt, 2005; Hooks, 2021; Seidler et al., 2022).

The analyzed research emphasizes that when some men encounter professionals who show empathy, with therapeutic interventions that do not focus on medicalization and with communication adapted to their needs and experiences, they achieve greater involvement in their processes of transformation to overcome their problems. In this way, help-seeking is reframed as a determinant of health and as a fundamental skill within processes of self-care and responsibility for men, eliminating stigma and comparisons that frame help-seeking as a behavior that makes men weaker. For this reason, future models of psychotherapeutic interventions with men may benefit from moving beyond simple modifications of existing models. More comprehensive and gender-responsive approaches appear warranted, fostering a critical perspective on traditional and androcentric frameworks, and introducing a transversal gender approach that could provide more cutting-edge techniques, theories, and models, potentially strengthened by the inclusion of studies on egalitarian masculinities.

This proposal is made because the most recent research emphasizes the need to develop gender-sensitive therapeutic devices capable of questioning and making masculine mandates visible, while it is very important not to pathologize or stigmatize them, considering that the majority of men in our societies have been socialized under these types of mandates (Camacho-Ruiz et al., 2024; Connell & Messerschmidt, 2005; Hooks, 2021; Seidler et al., 2022). For this reason, it is necessary to legitimate different alternatives for emotional expression, giving value to care in social and interpersonal relationships, also among men within peer groups, especially during childhood and adolescence, since changes are currently being observed in some parental relationships with their children. Increasing this type of relationship aims to enhance empathy and reciprocity. It is also fundamental for men in order to increase self-knowledge, as it helps to explore the affective domain in depth in relationships both with others and with themselves. For this purpose, it is absolutely necessary to start from a multifactorial analysis with the application of different interdisciplinary approaches, and it is also imperative to maintain an intersectional approach that is responsive to new needs and to the different sociocultural structures that operate in the configuration of masculine subjectivity.

The main limitations of this study include the geographic concentration of evidence in English-speaking countries, which underscores the need for studies conducted in diverse sociocultural contexts (e.g., Southern Europe, Latin America, and Asia) to strengthen external validity and to clarify how local gender norms, health systems, and social inequalities shape men’s distress, help-seeking, and treatment engagement. In addition, restricting the search to publications in English and Spanish may have introduced language bias, potentially excluding relevant evidence from other linguistic contexts and limiting the comprehensiveness of the review. While we searched three major databases (PubMed, Scopus, and Web of Science), the omission of subject-specific sources such as PsycINFO (and potentially CINAHL) may have limited the breadth of evidence captured, particularly within psychology- and nursing-focused literature. Moreover, we did not include grey literature or undertake hand-searching of specialist journals or forward/backward citation tracking, which may have further constrained the breadth of the evidence captured. Furthermore, the predominance of heterosexual samples highlights a critical gap in understanding how masculinities are lived among LGTBIQ+ and non-binary populations (i.e., subordinate masculinities), and how these experiences intersect with stigma, minority stress, and pathways into care. Future research should purposively recruit and analyze these groups rather than treating them as residual categories. In addition, the absence of older men and men facing extreme socioeconomic vulnerability (i.e., marginalized masculinities) limits life-course and equity-oriented clinical recommendations. Longitudinal and intersectional designs are required to examine cohort effects, cumulative disadvantage, and barriers related to cost and service navigation. Finally, we acknowledge that a formal quality appraisal or risk-of-bias assessment of the included studies was not conducted; therefore, the findings and clinical implications should be interpreted with appropriate caution. Clinically, these gaps suggest the need to develop and evaluate gender-sensitive and context-responsive interventions, supported by workforce training, that can be adapted to heterogeneous masculinities and structural constraints across settings.

In addition, further research is needed to clarify the impact of COVID-19 on men’s mental health and help-seeking, particularly in relation to disruptions in mental health service delivery, shifts towards digitally mediated care, and potential changes in gendered norms around vulnerability and self-reliance. Studies should also examine whether these effects were unevenly distributed across social groups (e.g., by socioeconomic position, ethnicity/racialization, migration status, age, and sexual orientation), and how intersecting structural constraints shaped access, acceptability, and continuity of care. Longitudinal and comparative designs (pre-, during-, and post-pandemic) would be especially valuable for distinguishing pandemic-related effects from broader secular trends in men’s help-seeking.

To conclude, based on the studies analyzed, the gender perspective in interventions with men should not be considered a mere ideological or methodological addition; it must be an indispensable dimension in order to understand and intervene in mental health therapeutic processes with men. Incorporating a gender perspective not only improves the effectiveness of intervention, but it also allows for questioning gender norms that perpetuate the exclusion and emotional repression of men, which have been viewed as inherent and natural both in mental health intervention practices and in the construction of theory and scientific discourse. The results obtained in the current narrative review show two clear issues: on the one hand, they invite reflection on the diagnostic and therapeutic frameworks that are currently commonly used, recommending the incorporation of an intersectional approach that considers gender as a key determinant of health. Moreover, they highlight the relevance of promoting specialized training not only in gender, but also in new ways of intervening with men from a gender perspective, emphasizing the inclusion of egalitarian masculine narratives, the promotion of an ethics of care, the development of help-seeking capacities, relational competencies, and the deconstruction of gender stereotypes.

### Clinical and Practice Implications

From a clinical and service-delivery perspective, the findings support the need for gender-sensitive and context-responsive mental health care for men. In practical terms, this requires strengthening the capacity of professionals to recognize how hegemonic masculinity norms (e.g., self-reliance, emotional control, invulnerability) shape men’s distress presentation, delay help-seeking, and contribute to early disengagement. Training programs for mental health providers should therefore prioritize (I) identifying gendered expressions of depression, anxiety, and psychological distress, including externalizing symptoms (e.g., irritability, anger, substance use, risk-taking); (II) communication strategies that reduce shame and defensiveness while maintaining a non-stigmatizing stance; and (III) techniques to stablish therapeutic alliance with men who may initially present with low readiness for emotional disclosure. Applied formats such as case-based learning, simulated consultations, and role-play can support clinicians in translating these principles into practice, particularly when addressing ambivalence, avoidance, or perceived threats to identity during early sessions.

Gender-sensitive approaches should also be reflected in assessment and formulation. Beyond symptom severity, clinicians may benefit from incorporating brief structured tools to explore help-seeking attitudes, self-stigma, and conformity to restrictive masculinity norms, as well as routinely assessing key engagement risks (e.g., prior negative experiences with care, perceived loss of autonomy, low mental health literacy, limited informal support). Embedding these domains in the clinical formulation can help clinicians collaboratively link men’s presenting difficulties to the interaction between individual coping strategies and gendered social expectations, rather than framing distress exclusively as personal weakness or non-compliance.

At the level of therapeutic process and intervention design, services may improve engagement by offering approaches that are both emotionally attuned and action-oriented. In many cases, interventions that combine clear goals, collaborative problem-solving, and skills-based strategies with gradual work on emotional awareness may be especially acceptable to men who experience emotional disclosure as threatening or unfamiliar. Approaches such as Acceptance and Commitment Therapy (ACT) or compassion-focused methods may be useful when delivered in ways that normalize emotional difficulty, emphasize functional coping, and connect change efforts to personally valued roles (e.g., fatherhood, partnership, work functioning). Integrating motivational interviewing techniques may further strengthen adherence by aligning therapeutic tasks with men’s own values and autonomy, reducing drop-out associated with perceived judgment or loss of control.

Service designers can also reduce barriers by implementing male-friendly pathways into care. Practical modifications include simplifying intake procedures, minimizing bureaucratic obstacles, offering flexible appointment formats (including digital or outreach-based contacts), and improving signposting through clear and user-oriented service navigation materials. Where feasible, delivering sessions in community-based or familiar environments (e.g., primary care settings, workplaces, community centers) may reduce perceived stigma and increase uptake. Attention to the therapeutic environment—privacy, non-clinical tone, and relational safety—may further support emotional openness, particularly for men whose engagement is shaped by concerns about judgment or status loss.

Notably, gender-sensitive practice should be intersectional, recognizing that men’s barriers and needs differ by age, socioeconomic position, ethnicity, migration history, and sexual and gender diversity. Tailored approaches are particularly warranted for men facing severe socioeconomic vulnerability, as structural constraints such as cost, limited access, and service fragmentation can compound stigma-related barriers. Similarly, LGTBIQ+ and non-binary individuals may experience additional minority stress and distinct patterns of marginalization that require culturally competent, affirming care rather than reliance on heteronormative assumptions. Finally, prevention-oriented actions—including stigma-reduction campaigns and early educational programs promoting emotional literacy and egalitarian masculinities—may complement clinical strategies by reshaping norms that impede help-seeking. Across all levels, gender-sensitive interventions should be evaluated and iteratively refined using patient feedback and implementation outcomes to ensure they remain effective and adaptable across sociocultural contexts.

## 5. Conclusions

To sum up, when the included studies are analyzed as a whole, they show that, in help-seeking processes, not only does living under the norms or values of hegemonic masculinity hinder the initiative to seek support, but these norms are also key to perpetuating a model of masculinity that rejects vulnerability, promotes emotional illiteracy and isolation, and has a significant impact on the mental health of many men.

The current narrative review shows how hegemonic or traditional masculinity, understood as a set of socially and culturally constructed gender norms or mandates, profoundly influences how men manage, interpret, and experience their mental health. This way of experiencing this phenomenon is not the same for all men; the study indicates that masculinity cannot be viewed in the singular, as there are diverse ways of living it, although the majority of men continue to express their masculinity through traditional mandates or norms that persist over time. These mandates are mainly based on the perception that vulnerability is equivalent to weakness; that a man must solve problems on his own and be self-sufficient; that he should not express his emotions publicly; that rationality should prevail over emotionality; and that affectivity between men should not be expressed, except for emotions related to strength or competitiveness, such as anger. This social and cultural construction of masculinity has consequences, specifically for the subjective construction of masculine identity among many men, which directly influences professional help-seeking processes aimed at addressing mental health-related problems.

Another important line highlighted by the current research is the need to introduce, across different clinical, educational, and community social resources, a transversal gender approach, since some studies show that when these approaches are incorporated, more favorable actions are generated for emotional exploration and expression, the acceptance of vulnerability, the overcoming of stigma, and the incorporation of an ethics of care that promotes processes of co-responsibility and self-care. The need to introduce role models of alternative and egalitarian masculinities is emphasized, making other models of masculinity visible within socialization processes, as well as designing therapeutic strategies based on these models of masculinity. These types of models are considered necessary to address the psychological distress of many men experience in different ways and context.

## Figures and Tables

**Table 1:** Study Characteristics and Participant Diversity Indicators.

Authors/Year	Objective	Sample (Gender and Age) *	Country/Setting	Design/Methodology	Main Findings	Main Limitations
Lynch et al. (2018).	To explore the barriers faced by young men aged 18 to 24 in seeking professional help for mental health problems, and to analyze the solutions they propose that are relevant to their lived realities. This aims to improve the mental health of this demographic group, enhance meaningful interventions, and contribute to suicide prevention measures.	The study sample consisted of 17 young men aged between 18 and 24 years, residing in County Donegal, in northwestern Ireland. Geographical Background: Participants were selected from urban, rural, and Irish-speaking areas within County Donegal, North West Ireland. Ethnic Background: The sample included individuals of various ethnicities, such as Irish, Northern Irish, Irish American, Greek American, Scottish, and Chinese.	The study was conducted in County Donegal, located in the North West of Ireland. Recruitment took place at a local youth service in County Donegal, which serves approximately 5000 young people across the county.	Qualitative constructivist design aimed at exploring barriers and solutions to professional mental health help-seeking. Data were collected through two focus groups with six participants each to capture social dynamics, and five face-to-face individual interviews to obtain personal and confidential perspectives. A semi-structured interview guide was applied, beginning with open questions and followed by more focused prompts on personal experiences and proposed solutions.	The main findings of the study identify seven key barriers to young men’s help-seeking professional support: peer acceptance, personal challenges, cultural and environmental influences, self-medication with alcohol, negative perceptions of professional help, fear of homophobic responses, and traditional masculine ideals.	Recruitment difficulties: The sensitive nature of the topic limited participant availability. Specific sample: Most participants were involved in a youth center, thereby excluding the voices of young men less willing to discuss mental health issues and of groups such as young men from the Irish Traveler community. Limited generalizability: The findings are not applicable to other countries, although parallels may be drawn with contexts characterized by a strong Catholic heritage. Potential bias: Despite measures such as member checking and reflexivity, latent biases on the part of the lead researcher may remain.
Wilson et al. (2022).	The primary objective of the study is to examine parents’ and teachers’ perceptions of the development of masculinity within a private all-boys school context, as well as their views on priorities for school-based initiatives designed to support students toward positive trajectories of masculine identity development. In addition, the study seeks to identify potential links with the promotion of mental health through changes in masculine norms.	The study sample consisted of 16 participants, including 10 parents (6 women and 4 men, all with a child in Year 11) and 6 teachers (3 women and 3 men), recruited from a high-fee private all-boys school. Participants ranged in age from 32 to 73 years, with a mean age of 49.19 years and a standard deviation of 10.35 years. Birth Country: 56.2% of participants (9 individuals) were born in Australia, while 43.8% (7 individuals) were born overseas (United Kingdom: 5 participants, New Zealand: 2 participants). Sexual Orientation: All participants identified as heterosexual.	The study was conducted in Melbourne, Australia, specifically at a high-fee independent all-boys’ grammar school. The setting is significant as it focuses on a single-sex private school environment, which is often associated with unique cultural and social dynamics influencing masculinity development.	Qualitative research design based on individual semi-structured interviews aimed at exploring attitudes toward masculine identity development within a private all-boys’ school context. Data were collected through interviews guided by a discussion schedule aligned with the research question, conducted both in person at the school and by telephone. Interviews were audio-recorded, transcribed verbatim, and checked for accuracy. Data analysis followed Braun and Clarke’s thematic analysis framework, using an inductive approach driven by the interview material and conceptually informed by Connell’s masculinities framework. Themes were developed through constant comparative methods, with the research team reaching consensus on interpretations and illustrative extracts.	Students adjust their behavior according to the social context, adopting “masks” to conform to traditional masculine norms. Sporting culture and “blokey” humor reinforce traditional masculine norms. Such schools can both challenge and reinforce traditional masculine norms. The findings highlight the importance of engaging parents and teachers in school-based initiatives to promote healthy masculinities and improve students’ mental health.	Limited sample: The study focused on a single high-fee private all-boys school, which restricts the generalizability of the findings to other contexts, such as lower-resourced or coeducational schools. Homogeneous participants: All participants identified as heterosexual, limiting the applicability of the findings to parents and teachers from sexual minority groups. Potential response bias: Participants with a particular interest in the topic may have been more likely to take part, and some may have censored their responses due to fear of judgment. Cis-heteronormative focus: The prevailing perspective may have limited the exploration of diverse masculinities, such as those of transgender or non-binary individuals. These limitations highlight the need for more inclusive and diverse research.
Kwon et al. (2023).	The primary objective of the study is to better understand the reasons men give for disengaging from mental health services and to identify factors that could facilitate their re-engagement with the care system.	The study sample included 73 men who participated in a national survey conducted by Lived Experience Australia (LEA). These participants were users of mental health services in Australia. It is noted that ethnic, cultural, and sexual minority populations were underrepresented in the sample.	The study was conducted in Australia and included participants from all states and territories: Victoria, South Australia, New South Wales, Queensland, Tasmania, Western Australia, the Northern Territory, and the Australian Capital Territory. Participants lived in a range of settings, from capital cities to regional centers and remote towns. Data were collected via a national survey administered by Lived Experience Australia (LEA), an Australian mental health consumer and carer advocacy organization.	Survey Structure: The survey contained 42 questions, including both quantitative and qualitative questions. Responses to Questions 35–42 were excluded as they focused on private mental health services. Qualitative Analysis: Researchers conducted iterative readings of survey responses to identify themes. They used Seidel’s (1998) “Noticing, Collecting, and Thinking” qualitative data analysis process to categorize and refine themes. Two main themes and seven subthemes were identified.	Reasons for men’s disengagement: These include a lack of autonomy, professionalism, and authenticity within services, as well as systemic barriers such as inconsistency, insufficient follow-up, and accessibility issues. Factors that may facilitate re-engagement: Key factors include clinician-initiated reconciliation, support from community workers and peers with lived experience, and the simplification of processes for re-entering services.	Small sample size: Only 73 men participated. Limited survey period: The survey was open for only three weeks. Lack of diversity: Ethnic, cultural, and sexual minority groups were underrepresented. Absence of specific data: Diagnostic information was not collected, nor were data disaggregated for young men aged 18–24 years. Limited generalizability: The findings may not be applicable to other countries or contexts.
Stewart and Harmon (2004).	The primary objective of the study is to challenge traditional beliefs about angry men, who are frequently diagnosed with antisocial personality disorder (ASPD) and often regarded as untreatable cases. The article seeks to explore similarities between ASPD and borderline personality disorder (BPD), highlighting their association with childhood trauma and proposing a re-evaluation of diagnosis and treatment approaches.	The study includes a detailed case study of a 31-year-old man referred to as “John” (a pseudonym), who presented with aggressive behaviors, a history of childhood trauma, mental health difficulties, and challenges in his interpersonal relationships. This case is used to illustrate the challenges and therapeutic approaches involved in the treatment of men with aggressive behaviors and borderline personality disorder.		The research is divided into two parts: (I) Literature Review: The first part discusses existing research on the causes of aggressive and antisocial behaviors in men, the role of childhood trauma, and the similarities and differences between Antisocial Personality Disorder (ASPD) and Borderline Personality Disorder (BPD). It also critiques biases in diagnosis and treatment approaches for these disorders. (II) Case Study: The second part presents a detailed case study of “John,” a 31-year-old male with a history of childhood trauma, aggressive behaviors, and self-harm. The case study illustrates the challenges and strategies involved in engaging and treating men with these issues.	The main findings indicate that men with aggressive behaviors and antisocial personality disorder (ASPD) can benefit from structured therapeutic approaches that consider their traditional values and childhood trauma. The case study demonstrates that, with connection, clear boundaries, and ongoing support, significant improvements in mental health and behavior are achievable.	The main limitations include diagnostic bias between ASPD and BPD, a lack of differentiation between antisocial behaviors and antisocial personality traits, and the prevailing perception of ASPD as untreatable. In addition, traditional therapeutic approaches are often incompatible with traditional masculine values, which may hinder men’s engagement in treatment.
Boettcher et al. (2019).	The primary objective of the study is to contribute to the theoretical understanding of work-related mental health experiences among men and to explore opportunities for employers to provide gender-sensitive support for men’s mental health. Specifically, the study examines how masculine role norms influence work-related stress and mental health, using narratives from men employed in male-dominated occupations.	The study sample comprised 18 men employed in male-dominated occupations, selected from a larger pool of 37 participants. These men were working full-time, and the majority appeared to be Caucasian and based in Canada. The sample in this study was relatively homogeneous, with the majority of participants appearing to be Caucasian and of middle to upper socioeconomic status. Additionally, all participants were employed full-time, and the sample over-represents the energy sector due to the geographic location of the lead investigator and data collection team. Perspectives from men of diverse socioeconomic and cultural backgrounds, as well as part-time, seasonal, or precariously employed men, were not captured.	The study was conducted in Canada, with participants recruited through various avenues such as municipal Kijiji directories, emails targeting male-dominated work sectors, and Canadian Immigration Services. The sample over-represents the energy sector, which has been recently affected by economic challenges in Canada.	For the qualitative component, a narrative approach was employed to collect and analyze stories from participants about workplace stress, coping strategies, and employer support. The analysis was guided by Braun and Clarke’s contextualist approach to qualitative thematic analysis, balancing descriptive data with theoretical concepts related to masculine role norms. The study also used Addis and Mahalik’s (2003) typology to identify and code instances of injunctive, descriptive, and cohesive masculine role norms. Data collection involved focus groups and individual interviews. Interviews were semi-structured, lasting between 30 and 130 min, with an average length of 44 min.	The main findings show that masculine role norms influence men’s work-related stress and mental health. These norms are divided into three types: Descriptive norms: Men adjust their work behavior according to that of their peers, which can normalize overwork and make help-seeking more difficult. Injunctive norms: Internal beliefs about what men “should” do at work, generating anxiety and questioning of personal worth during periods of low productivity. Cohesive norms: Leaders model and communicate performance expectations, often contradicting mental health support policies. These norms reinforce behaviors that may compromise men’s mental health in the workplace.	Sample homogeneity: Most participants were Caucasian and from a middle-to-upper socioeconomic background, which limits the representation of men from diverse backgrounds. Participant self-selection: Men interested in discussing workplace mental health may have biased the results. Lack of occupational diversity: Only full-time employed men were included, excluding the perspectives of part-time or precariously employed workers. Overrepresented sector: A predominance of participants from the energy sector, which was affected by the economic recession. Secondary data: The analysis was based on responses to general questions about work-related stress, rather than specifically on masculine role norms.
Edwards et al. (2017).	The primary objective of the study is to analyze differences between male and female caregivers using data from the 2009 Behavioral Risk Factor Surveillance System (BRFSS).	The study sample was drawn from the 2009 Behavioral Risk Factor Surveillance System (BRFSS), which included 421,215 participants from all 50 U.S. states. The sample includes both male (32.5%) and female (67.5%) caregivers, allowing for gender comparisons in caregiving experiences and health outcomes. Race/Ethnicity: Participants were categorized into four groups: White Non-Hispanic, Black Non-Hispanic, Hispanic, and Other Non-Hispanic or Multi-Racial. Age: Respondents were divided into five age categories: 18–29, 30–39, 40–49, 50–59, and 60 years or older. Education Levels: Education was categorized into four levels: Less than High School, High School Graduate, Some College, and College Degree or Higher. Disability Status: Approximately one-quarter of caregivers self-identified as disabled.	The study was conducted in the United States, encompassing all 50 states, the District of Columbia, and three U.S. territories (Guam, Puerto Rico, and the Virgin Islands). The data was collected through the 2009 Behavioral Risk Factors Surveillance System (BRFSS), which is the largest annual telephone health survey in the U.S.	Study Design: The study utilized a cross-sectional design, analyzing data from the 2009 Behavioral Risk Factors Surveillance System (BRFSS), a large-scale annual telephone health survey conducted in the United States. Sampling Method: The BRFSS employed random-digit dialing methodology and weighting to ensure the sample was representative of the demographic characteristics of each state. Key Variables: (I) Independent Variables: Gender, social/emotional support, age, race, marital status, education, smoking status, disability status, and leisure-time physical activity. (II) Dependent Variables: Health-related quality of life (HRQOL), including physically and mentally unhealthy days, life satisfaction, and general health. For data analysis, the study used SPSS Version 22 to analyze the data, accounting for the complex sampling design of the BRFSS.	Gender distribution: Two-thirds of caregivers were women. Unhealthy days: Women reported more physically and mentally unhealthy days than men. Social support: Men were more likely to report rarely or never receiving social support, but the impact of social support on quality of life was stronger for men than for women. Associated factors: Age, education, and social support were associated with fewer unhealthy days, whereas smoking and physical inactivity were associated with more unhealthy days.	Caregiving period: The BRFSS question only covered the previous month, excluding caregivers outside that period. Telephone coverage: Only individuals with landline telephones were included, which may have underrepresented younger adults and those with fewer resources. Measurement of social support: A single item was used, which is less precise than a full scale. Marital relationship: The quality of marital relationships was not assessed, which may have influenced the results.
Seidler et al. (2021b).	The primary objective of the study is to examine perceived barriers—both attitudinal and structural—that hinder men’s access to mental health services. In addition, the study seeks to analyze the associations between these barriers and men’s intentions to seek or not seek treatment for their mental health concerns.	The study sample consisted of 778 men who reported experiencing a mental health concern and were not receiving treatment at the time. Participants ranged from 18 to 77 years old, with a mean age of 35.63 years. Sexual Orientation: 84% identified as heterosexual. Ethnicity: 70% identified as Caucasian. Education: 72% had some post-secondary education. Employment Status: 69% were currently employed. Substance Use: 30% reported struggling with problematic alcohol or drug use. These indicators highlight a range of demographic and personal characteristics within the sample.	The study was conducted in an international setting, with participants recruited through the HeadsUpGuys website, a Canadian online resource for men with depression. Ethical approval was granted by the University of British Columbia in Canada. Additionally, the authors are affiliated with institutions in Australia and Canada, indicating a focus on these regions.	Cross-sectional quantitative survey design aimed at examining barriers to accessing mental health services among men with current mental health concerns. Data were collected through an online questionnaire administered after informed consent. The survey assessed perceived structural and attitudinal barriers to mental health services using a standardized barriers scale, as well as levels of psychological distress measured with a brief screening instrument. Data analysis included descriptive statistics, chi-square tests, t-tests, and logistic regression analyses to examine associations between perceived barriers and intentions to seek treatment, as well as to identify key predictors of not wanting to access mental health care.	Common barriers: The most frequent barriers were attitudinal, such as “many people feel sad and depressed” (80%) and “I need to solve my own problems” (73%), and structural, such as “I do not know what to look for in a psychotherapist” (80%) and “I cannot afford psychotherapy” (72%). Differences by treatment intention: Men who did not want treatment were more likely to doubt the effectiveness of psychotherapy, not disclose their emotional state to their physician, and prefer to solve their problems on their own. Predictive factors: Attitudinal barriers, such as the need to solve problems independently, were significant predictors of not wanting treatment.	Cross-sectional design: The data were based on a single survey, which limits the ability to examine changes over time. Predefined barriers: Not all possible barriers were included, which may have led to the omission of important factors. Specific sample: Participants had already recognized a mental health concern and were recruited through an online resource, limiting generalizability to men who do not seek help or lack awareness of their mental health. Prior interaction: It is unclear whether use of the HeadsUpGuys website influenced participants’ responses.
Tibubos et al. (2022).	The primary objective of the study is to develop and validate a brief measure to assess gender expression using a shortened version of the Personal Attributes Questionnaire (PAQ-8), and to examine its associations with mental distress in the German population in 2006 and 2018. In addition, the study aims to explore how expressions of femininity and masculinity contribute to the mental health gap between men and women.	The study sample includes data from two representative studies conducted in Germany in 2006 and 2018. Participant information is detailed below: Year 2006: Total participants: 2507 individuals. Mean age: 48 years (standard deviation: 18.1). Representativeness: The sample was representative of the general German population in terms of age, sex, and educational level. Year 2018: Total participants: 2516 individuals. Mean age: 48 years (standard deviation: 17.6). Representativeness: Similar to the 2006 study, with comparisons made using census data from the German Federal Statistical Office. The sample was representative of the German population in terms of educational level. Household income was adjusted for household size and stratified into tertiles.	The study was conducted in Germany and involved representative survey data collected from the general German population. The setting included 258 non-overlapping sample point regions across the country.	Cross-sectional study with data collected in 2006 and 2018 aimed at developing and validating a short measure of gender expression (PAQ-8) and examining its association with mental distress over time. Representative German population samples were obtained using a random route procedure across 258 sampling regions, with household and respondent selection ensuring representativeness by age, sex, and education, confirmed against national census data. Data collection involved face-to-face interviews and self-administered questionnaires. Gender expression was assessed using the Personal Attributes Questionnaire, from which the PAQ-8 was derived, while mental distress was measured with the PHQ-4; socio-demographic variables were included as covariates. Statistical analyses combined item analysis, principal component analysis, confirmatory factor analysis, correlation analyses, MANOVA, and multiple regression to validate the scale and test associations with mental distress. Ethical approval was granted by the institutional review board of the University of Leipzig, and informed consent procedures were applied.	PAQ-8 validity: The brief version of the questionnaire (PAQ-8) is a valid measure for assessing gender expression, with good internal consistency and the ability to distinguish between masculinity and femininity. Changes in gender expression: Between 2006 and 2018, femininity increased among women and decreased among men, while masculinity remained stable. Association with mental distress: Higher levels of femininity and masculinity were associated with lower mental distress. Gender expression was a better predictor of mental health than biological sex. Impact of time: An increase in gender differences in the expression of femininity between men and women was observed over time.	Lack of non-binary categories: Gender options beyond male and female were not included, excluding individuals with diverse gender identities. Limited aspects of gender: Only gender expression (masculinity and femininity) was assessed, leaving out other dimensions of gender. Cross-sectional nature: The data were derived from cross-sectional studies, preventing the establishment of causal relationships. Selection bias: Non-participation by some individuals may have introduced bias into the sample. Androgynous gender not assessed: Although mentioned in theory, androgynous gender was not included as a separate category in the analysis.
Sharp et al. (2023).	The primary objective of the study is to explore the influence of masculinities and Australian culture on men’s mental health.	The study sample included 43 men (mean age: 50.7 years, SD: 13.8) residing in the Greater Sydney and Blue Mountains regions of New South Wales, Australia. The research explored the connections between Australian masculinities, culture, and men’s mental health within the Australian context. The study included several indicators of sample diversity among the 43 male participants: Education: The sample included individuals with varying levels of education: Less than Year 12 or equivalent: 2%. Year 12 or equivalent: 5%. Associate diploma or certificate: 21%. University degree: 72%. Marital Status: Participants had diverse marital statuses: Single/Never married: 16%. Married/Domestic partnership: 63%. Widowed: 2%. Divorced: 14%. Separated: 5%. Employment Status: Participants had different employment situations: Full-time work: 58%. Part-time work: 26%. Retired: 9%. Unemployed: 5%. Household Income: Annual household income varied: Less than $25,000: 9%. $25,000–$34,999: 2%. $35,000–$49,999: 2%. $50,000–$74,999: 9%. $75,000–$99,999: 12%. $100,000–$124,999: 16%. More than $125,000: 40%. Prefer not to say: 7%. Residential Background: 77% of participants had lived in Australia their entire lives. 23% had immigrated to Australia or lived abroad for a significant portion of time, with an average of 28.8 years in Australia. Geographical Location: 74% lived in urban areas. 26% lived in suburban areas.	The study was conducted in Australia, specifically focusing on men living in the Greater Sydney and Blue Mountains regions of New South Wales.	The study employed a qualitative design using five focus groups conducted between July and November 2019. Participants were recruited from the Greater Sydney and Blue Mountains regions of New South Wales, Australia, as part of a larger study aimed at developing a gender-transformative mental health promotion intervention for men. The focus groups consisted of 6–10 men per group, with a total of 43 participants. A semi-structured focus group guide was used to explore participants’ perceptions and experiences of masculinity, culture, and mental health. Open-ended questions encouraged dialogue, storytelling, and interaction without leading responses. The discussions were audio-recorded and transcribed verbatim. The data were analyzed using inductive thematic analysis, following Braun and Clarke’s (2006) approach. Researchers identified patterns, coded text segments, and grouped codes into categories to create a framework reflecting the main topics discussed. The analysis was guided by Connell’s masculinities framework to explore the influence of cultural and gender factors on men’s mental health perspectives and behaviors.	History of strength and self-reliance: Traditional masculine norms in Australia, such as resilience and the “she’ll be right” attitude, make it difficult for men to seek help for their mental health. Social and geographical divisions: Although the culture of “mateship” promotes connection among men, it often limits emotional expression and genuine support. Masculine socialization and generational disconnection: Restrictive masculine norms, such as emotional repression, are transmitted across generations, but some men are renegotiating these norms to promote healthier behaviors.	Contextuality of masculinities: The findings do not reflect the experiences of all men in Australia due to local and cultural specificities. Participant self-selection: Men less willing to discuss their mental health may have been excluded. Lack of linkage between quotations and demographic characteristics: Specific participant data were not recorded for the quoted excerpts. Intersection with social determinants: Factors such as economic hardship, racism, or discrimination against marginalized groups were not sufficiently explored.
Mesler et al. (2022).	The primary objective of the study is to clarify the relationships between masculine gender role discrepancy, discrepancy stress, and traditional masculinity ideology in men’s health-related behaviors.	The study sample consisted of 459 men with a mean age of 34.07 years (SD = 12.06). Participants were recruited via the Prolific Academic platform and came from three countries: 56.6% from the United Kingdom, 29.4% from the United States, and 14% from Canada.	The study was conducted with participants from three countries: United Kingdom: 56.6% of the sample. United States: 29.4% of the sample. Canada: 14% of the sample.	Design: Two-part study with temporal separation of predictor and criterion variables to reduce common method variance. Measures: Masculine gender role discrepancy (5 items, α = 0.93), discrepancy stress (5 items, α = 0.84), traditional masculinity ideology (6 items, α = 0.89), and health behaviors assessed with the Multidimensional Health Behavior Index (MHBI; 7 subscales). Procedure: Time 1 assessed discrepancy, discrepancy stress, traditional masculinity ideology, gender, and age; Time 2 (3 days to 1 week later) assessed health behaviors and included a second attention check. Data Analyses: Moderated mediation using PROCESS Model 7 with 5000 bootstrap replications; spotlight analyses at the 16th, 50th, and 84th percentiles of the moderator; floodlight analysis using the Johnson–Neyman technique. Validity/Data Quality: Confirmatory factor analyses, Harman’s single-factor test, and bot and attention checks.	The main findings indicate that discrepancy stress negatively mediates the relationship between masculine gender role discrepancy and health-promoting behaviors, such as proactive safety, healthy social relationships, and stress management. In addition, discrepancy stress is positively associated with negative mental health outcomes. These effects are stronger among men with greater adherence to traditional masculinity ideology.	The main limitations include the use of an online sample, which may introduce attentional biases, a single method design that may produce common method bias, and small, albeit statistically significant, effect sizes. In addition, the use of multiple subscales may have increased the risk of Type I error.
Mostoller and Mickelson (2024)	To analyze the role of help seeking stigma as a mediating mechanism linking conformity to traditional masculine norms with men’s mental health status. To investigate how traditional masculine norms, internalized from an early age, influence perceptions of help seeking mental health problems and their impact on psychological well-being.	In the study, 326 men residing in the United States participated, aged between 18 and 75 years (mean age of 33 years). Among them, 12 identified as transgender men, who were also included in the analyses, as conformity to masculine norms may affect both cisgender and transgender men. Education: Some high school: 1.8%. High school/GED: 14.1%. Some college: 28.8%. College degree: 41.1%. Advanced degree: 14.1%. Race/Ethnicity: The sample included individuals from various racial and ethnic backgrounds: White: 73.9%. Black: 8.3%. Hispanic: 11.7%. Asian: 13.2%. American Indian: 1.5%. Pacific Islander: 0.6%. Other: 5.8%. Marital Status: Married: 26.1%. Cohabitating: 13.2%. Divorced/Separated: 4.0%. Widowed: 0.3%. Single/Never Married: 56.4%. Current Romantic Relationship: Yes: 50.9%. No: 49.1%. Income: Less than $20,000: 17.2%. $20,001–$40,000: 16.6%. $40,001–$60,000: 21.8%. $60,001–$80,000: 18.1%. $80,001–$100,000: 11.0%. $100,001–$120,000: 7.7%. More than $120,000: 7.4%. Religion: Atheist or Agnostic: 47.5%. Christian: 33.7%. Jewish: 2.8%. Muslim: 1.5%. Spiritual but not religious: 11.3%. Other: 3.1%. Political Identity: Republican: 14.7%. Democrat: 47.5%. Independent: 37.7% Sexual Orientation: Heterosexual: 85.0%. LGBQ: 15.0%. Gender Identity: The sample included 12 individuals who identified as transgender, and they were included in the analysis.	The study was conducted in the United States, and participants were recruited online via the Prolific platform. The sample consisted of 326 U.S. men aged 18–75 years old, representing various demographic backgrounds.	Design: Cross-sectional correlational study examining associations among conformity to masculine norms, help-seeking self-stigma, and mental well-being (perceived stress and depressive symptoms) in men. Participants and Recruitment: 326 U.S. men aged 18–75 recruited online via Prolific; four participants excluded for failing more than one of four attention checks; monetary compensation ($2.30 USD/£1.80 GBP). Survey Administration: Online survey (~15 min); measures presented in counterbalanced order to minimize response bias. Measures: Sociodemographics (age, race/ethnicity, income, education, sexual orientation, gender identity, relationship status, political affiliation, religiosity); conformity to masculine norms (CMNI-30); perceived stress (PSS-10); depressive symptoms (PHQ-9); political ideology (Pew Research Center Political Party Quiz); help-seeking self-stigma (SSOSH). Data Analysis: Sociodemographics examined as potential covariates (age controlled for perceived stress; age, political ideology, and romantic relationship controlled for depression); mediation analyses in SPSS using PROCESS Model 4 with help-seeking self-stigma as mediator; post hoc mediation models testing specific masculine norm facets (heterosexual self-presentation, power over women, self-reliance).	The main findings of the study indicate that conformity to masculine norms is associated with greater self-stigma related to help seeking, which in turn is linked to higher levels of perceived stress, but not to depression. In addition, specific aspects of masculinity, such as self-reliance, heterosexual presentation, and power over women, influence self-stigma and, in some cases, mental health.	Homogeneous sample: Predominance of White, cisgender, and heterosexual men, with low variability in the variables studied. Cross sectional design: This prevents establishing causality or examining changes over time. Lack of cultural diversity: Differences in masculine norms across ethnic and cultural contexts were not explored.

**Notes:** * Indicators of sample diversity are presented when reported by the original studies; otherwise, they are noted as not reported.

## Data Availability

The data that support the findings of this study are available on request from the corresponding author.
